# Hamartoma of mature cardiomyocytes presenting with atypical angina, ^18^F-fluorodeoxyglucose positron emission tomography uptake, and myocardial bridging: a case report

**DOI:** 10.1093/ehjcr/ytad077

**Published:** 2023-02-14

**Authors:** Giacomo Bianchi, Edoardo Zancanaro, Angela Pucci, Marco Solinas

**Affiliations:** Adult Cardiac Surgery Department, Ospedale del Cuore, Fondazione Toscana ‘G. Monasterio’, Via Aurelia Sud, 54100 Massa, Italy; Adult Cardiac Surgery Department, Ospedale del Cuore, Fondazione Toscana ‘G. Monasterio’, Via Aurelia Sud, 54100 Massa, Italy; Anatomic Pathology and Histopathology, Pisa University Hospital, Via Roma 67, 56100 Pisa, Italy; Adult Cardiac Surgery Department, Ospedale del Cuore, Fondazione Toscana ‘G. Monasterio’, Via Aurelia Sud, 54100 Massa, Italy

**Keywords:** Mature myocyte hamartoma, Primary cardiac tumour, Left ventricular mass, Myocardial bridging, Case report

## Abstract

**Aim:**

Hamartoma of mature cardiomyocytes is a rare tumor and the present case shows a complex diagnostic pathway to understand its nature and treatment options in a young patient. The myocardial bridge was also part of the clinical evaluation discovered during the diagnostic workout.

**Methods and results:**

A 27-year-old woman with atypical chest pain and a normal electrocardiogram received the diagnosis of neoformation of the interventricular septum with ^18^F-fluorodeoxyglucose (^18^F-FDG) uptake, and evidence of myocardial bridging on coronary angiography. On suspicion of malignancy, coronary unroofing and surgical biopsy was performed. The final diagnosis was hamartoma of mature cardiomyocytes.

**Conclusion:**

This case offers great insight into medical reasoning and decision-making process. Given the history of chest pain, the patient was evaluated for possible ischemic, embolic, or vascular causes. Given a left ventricular wall thickness ≥15 mm, hypertrophic cardiomyopathy (HCM) should always be suspected; nuclear magnetic resonance imaging is essential to distinguish between HCM. The magnetic resonance imaging is also critical in distinguishing HCM itself from tumoral phenocopies. To rule out a neoplastic process, ^18^F-FDG positron emission tomography (PET) was used. A surgical biopsy was performed, and the final diagnosis was completed after the immune-histochemistry study. A myocardial bridge was found during preoperative coronagraphy and was treated accordingly.

**Learning points**

- To evaluate the patient with suspected hypertrophic cardiomyopathy (HCM).
- To review the potential tumoural phenocopies of HCM and to establish a diagnostic pathway for differential diagnosis.
- To review the usefulness of second-line imaging (MRI and PET) to refine diagnosis along with potential pitfalls.
- To review the feature of the rare hamartoma of mature cardiomyocytes and the implications for its diagnosis and treatment.
- To review the incidental finding of myocardial bridging and clinicopathological correlations.

## Introduction

Hamartoma is broadly defined as a benign abnormal polyclonal proliferation of cells native to the affected organ. Hamartomatous lesions that are composed of mature muscle fibre bundles that occur in cardiac tissue have been called hamartoma of mature cardiac myocytes (HMCMs); HMCMs are rare tumours; only 47 cases have been described to date in both paediatrics and adults,^1^ and its diagnostic workup is a challenging scenario. The multidisciplinary approach has been the key to the final diagnostic findings and treatment option strategy.

## Timeline

**Figure:**
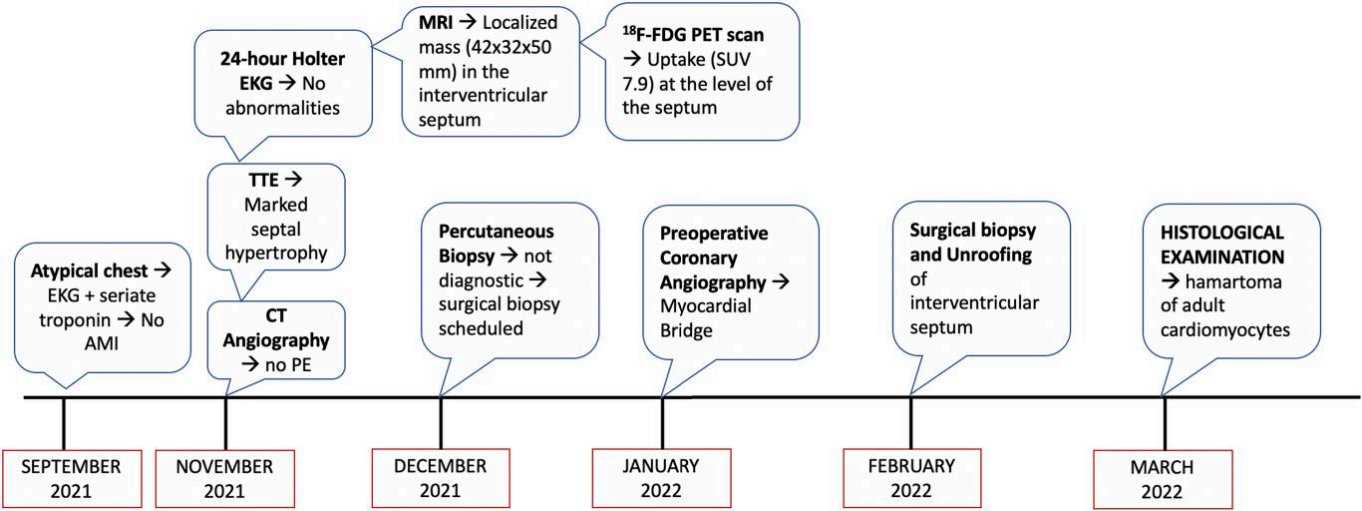


## Case presentation

A 27-year-old woman presented to the emergency department for atypical chest pain that had worsened in recent weeks. Past medical and family history was not significant. The electrocardiogram and seriated troponin assay were not suggestive of coronary syndromes. She had also undergone computed tomography with contrast medium (CT angiography) which excluded pulmonary embolism. A transthoracic echocardiogram (TTE) revealed the presence of marked septal hypertrophy (24 mm) without left ventricular outflow tract obstruction (LVOTO). The young woman was then referred to our centre for further characterization of the disease and possible treatment.

While an interventricular septal thickness of at least 15 mm, in the absence of other cardiac or systemic causes capable of leading to such hypertrophy, is considered sufficient for the diagnosis of HCM, there are, however, other clinical conditions that share the same phenotypic expression that must be ruled out^2,3^(*Table 1*). A 24-h Holter electrocardiogram, TTE, and nuclear magnetic resonance imaging (MRI) were therefore initially scheduled.

**Table 1:** Differential diagnosis in cardiomyopathies with morphological aspect of hypertrophic or restrictive phenotype

Primary HCM	Sarcoidosis
Primary RCM	Scleroderma
Amyloidosis	Pseudoxanthoma elasticum
Fabry’s disease	Carcinoid heart disease
Glycogen storage diseases	Primary and secondary heart tumours
Endomyocardial diseases	Drugs and radiations

HCM: hypertrophic cardiomyopathy; RCM: restrictive cardiomyopathy

**Figure 1:**
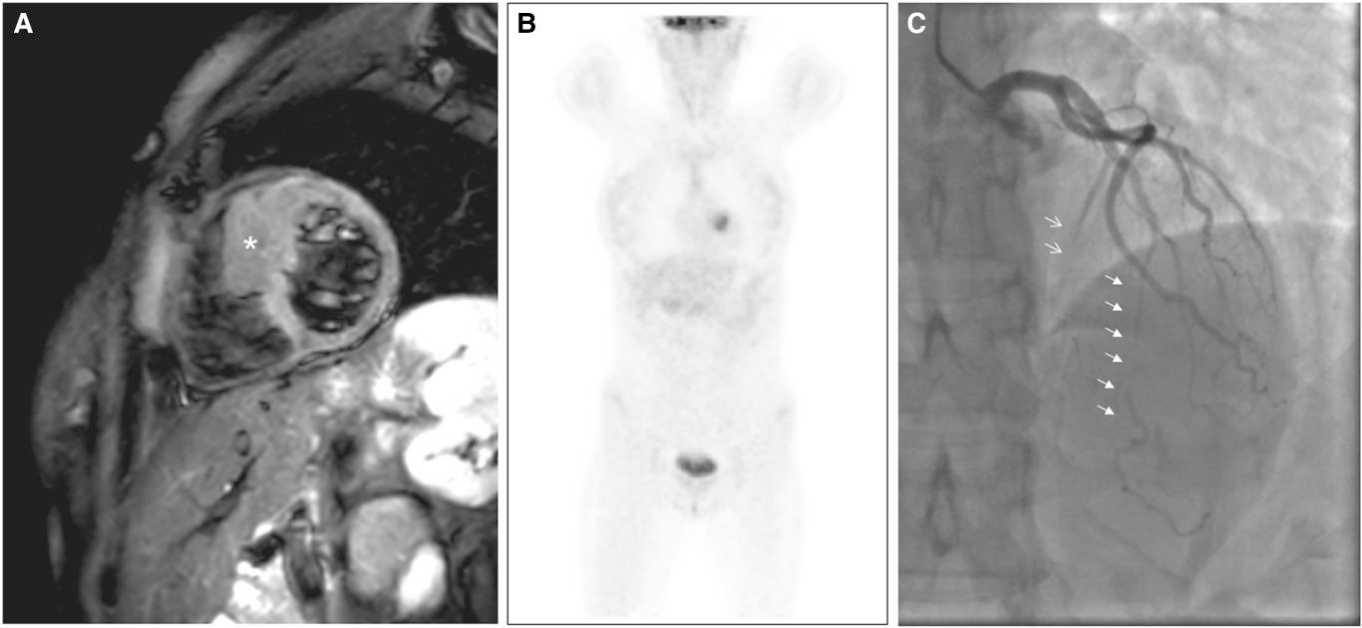
(*A*) T2* MRI image showing the mass (*) as a bulging of the interventricular septum with areas of inhomogeneous intensity. (*B*) ^18^F-FDG uptake of the mass, localized in the interventricular septum. (*C*) Coronary angiogram: ‘squeezing' of the first septal branch against the HMCM during systole (slant open arrows); ‘milking’ of the LAD due to a myocardial bridging (closed arrows) is also shown. HMCM: hamartoma of mature cardiac myocytes; LAD: left anterior descending artery.

The 24-h Holter electrocardiogram showed sinus rhythm with normal atrioventricular (AV) conduction and corrected QT interval. The MRI revealed a localized mass (42 × 32 × 50 mm) in the interventricular septum protruding into the right ventricle, in the context of overall increased septal thickness (*Figure 1A*). The tissue was isointense in T1 sequences compared with the surrounding myocardium and hypointense in its central core; at T2, short tau inversion recovery (STIR) appeared inhomogeneous and slightly hypointense; it appeared to have an inhomogeneous contrast enhancement in the early phase, along with an intralesional late enhancement. To rule out a tumoural phenocopies of mid-ventricular hypertrophic cardiomyopathy (HCM), the patient underwent a ^18^F-fluorodeoxyglucose positron emission tomography (^18^F-FDG PET) scan, which revealed uptake (SUV 7.9) at the level of the septum (*Figure 1B*); given the high suspicion of malignancy, a biopsy was deemed mandatory; unfortunately, it was not diagnostic. The patient was then referred for surgical biopsy of the mass. A second MRI performed 1 month later showed no growth of the nodular formation. On pre-operative examinations, coronary angiography revealed a myocardial bridge with a marked ‘milking’ effect at the middle level of the anterior descending artery (LAD); this effect was also present at the level of the first septal branch (*Figure 1C*). The surgical schedule was then aimed at unroofing the muscular bridge on LAD and surgical biopsy of the mass with right trans-atrial approach.

**Figure 2:**
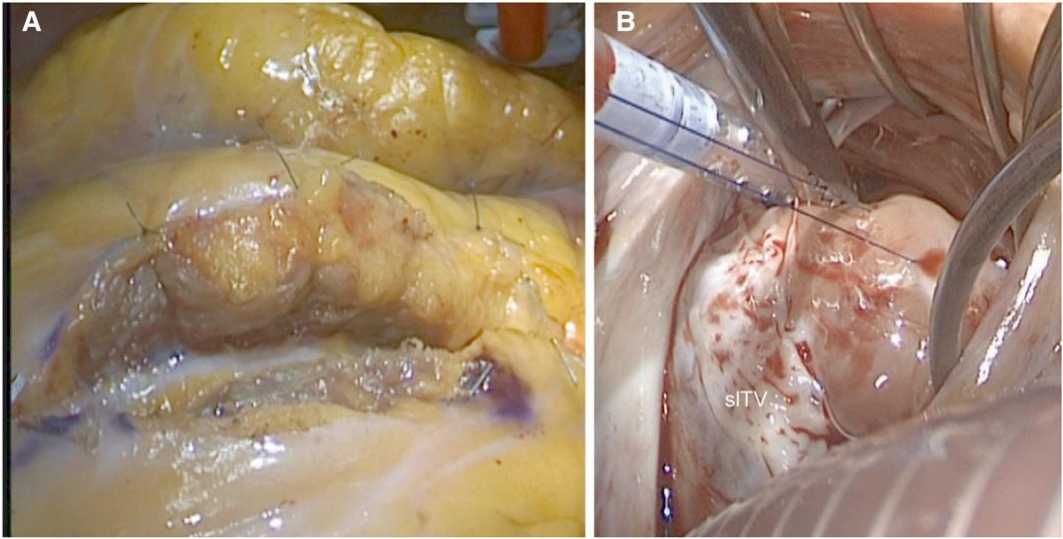
(*A*) Intraoperative image of the ‘unroofing’ of the LAD, laying 7 mm below a bridging muscle. (*B*) Intraoperative appearance of the mass, with the relationship with the subvalvular apparatus of the sLTV. LV: left ventricle; HMCM: hamartoma of mature cardiac myocytes; LAD: left anterior descending artery; slTV: septal leaflet of the tricuspid valve.

A median sternotomy was chosen for the procedure. On cardiopulmonary bypass (CPB) and cardioplegic arrest, the bridge myocardium was removed by muscle dissection (*Figure 2A*), sending a specimen for histology. After opening the right atrium, the mass was identified, protruding into the right ventricular outflow and partly involving the chordal apparatus of the tricuspid valve (*Figure 2B*). An extended specimen was then excised and sent for histology. Surgery subsequently proceeded according to routine.

The patient was transferred to the ICU and extubated after 4 h of mechanical ventilation. Transferred to the ward on the first postoperative day, she presented an event-free course and was discharged home in good general condition, free of symptoms.

The harvested tissue was processed according to standards for histologic examination, performing haematoxylin and eosin, Masson’s trichrome, and Perls’ and Congo Red staining on 3 μm sections. In adjacent sections, immunohistochemistry was performed for desmin, myoglobin, α-smooth muscle actin (1A4), myogenin (MyoD1), S100, and proliferating antigen Ki67.

**Figure 3:**
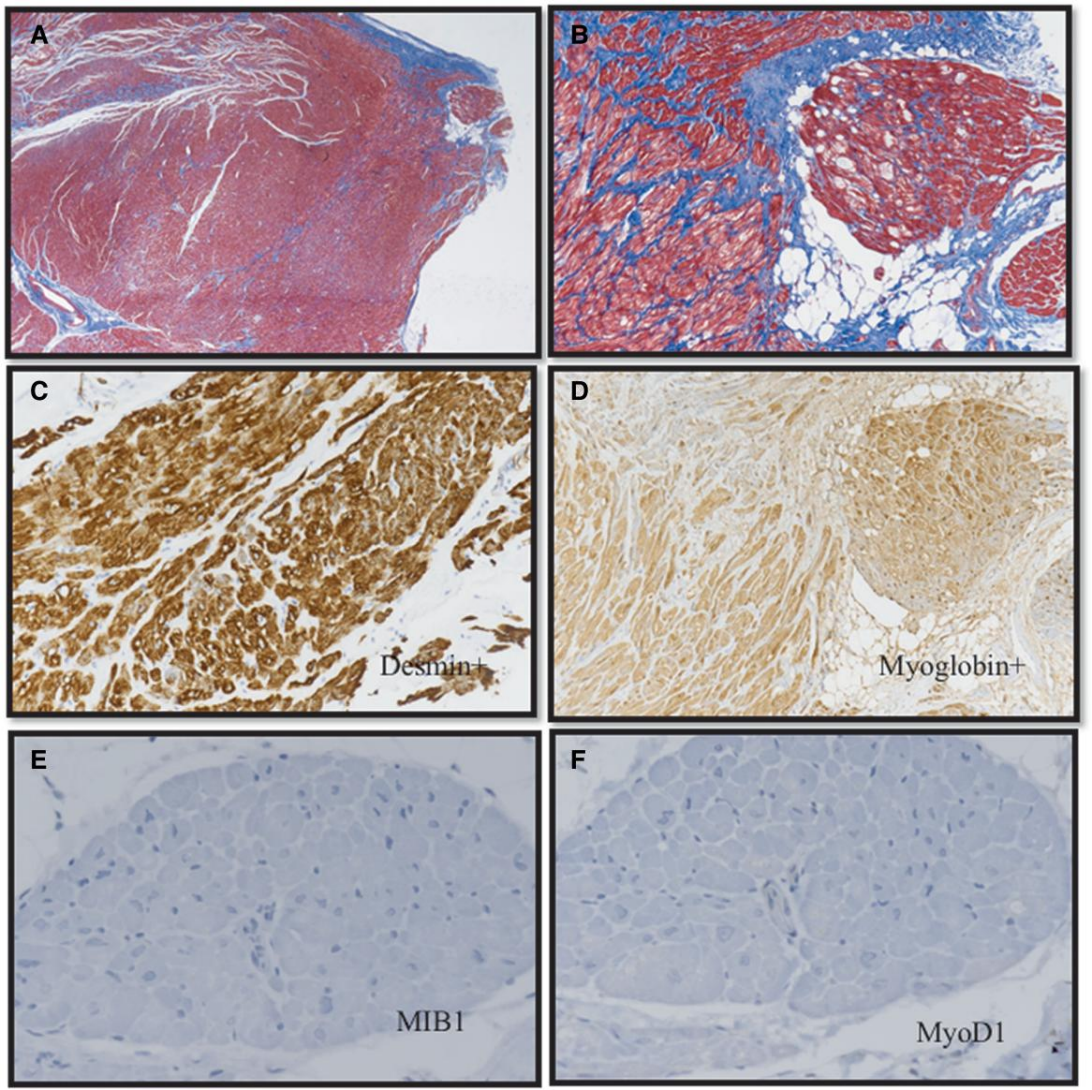
Surgical specimen of the mass; mature cardiomyocytes with hypertrophy and sarcoplasmic vacuoles, partially delimited by connective and adipose tissue (*A*–*B*), with positive immunoreaction for desmin (*C*) and myoglobin (*D*); no immunoreactivity is shown for proliferating Ki-67 index (*E*) or myogenin (MyoD1, *F*). *A*, *B*: Masson’s trichrome staining at magnification of ×4 (*A*) and ×10 (*B*); C–F: Immunoperoxidase with haematoxylin counterstaining (magnification ×10).

Histopathological investigations showed that the bulging mass had features (*Figure 3* panels *A*–*F*) compatible with a hamartoma of mature cardiomyocyte (HMCM), while the myocardial bridge consisted of normal cardiomyocytes.

The patient at 12 months is alive, in excellent general condition, and free of angina. Repeated serial echocardiograms at 1, 3, and 9 months after surgery show good biventricular function and stability of the ‘mass’ of the interventricular septum, which remains non-obstructive to left ventricular outflow.

## Discussion

This case offers great insight into medical reasoning and decision-making process. Given the history of chest pain, the patient was evaluated for possible ischemic, embolic, or vascular causes. Although the probability of a patient of this age having coronary artery disease is 4.2%±1.3%,^4^ serial troponin sampling was performed and left ventricular kinetics assessed on echocardiogram, ruling out ischemic cause. Computed tomography angiography scan of the chest had also excluded aortic dissection and pulmonary embolism. On echocardiographic examination, due to the presence of septal hypertrophy, a suspicion of hypertrophic cardiomyopathy (HCM) with limited expression had been raised.

Given the prevalence of HCM (1:500) in the general population, HCM should always be suspected in case of a left ventricular wall thickness ≥15 mm; nuclear MRI is essential to distinguish between HCM, athlete’s hypertrophy, metabolic and storage diseases, infiltrative processes (such as amyloid cardiomyopathy), and primary and secondary cardiac tumours.^5^ The MRI is also critical in distinguishing HCM itself from tumoural phenocopies. In this case, although the nodular formation might have been compatible with an expansive lesion, the late gadolinium enhancement (LGE) might have been suggestive of atypical HCM.^2^ To rule out a neoplastic process, ^18^F-FDG PET was used, which showed high radio drug uptake in that area (SUV 7.3)—a potentially dramatic picture since an SUV >4.3 is highly suggestive of primary malignant cardiac tumour.^6^ The most frequent primary cardiac tumours, such as sarcomas (angiosarcoma, rhabdomyosarcoma, and leiomyosarcoma), and forms such as cardiac lymphoma and amyloid light-chain (AL) amyloidosis have all been excluded by tissue characterization with MRI and degree of involvement of myocardial structures.^7–10^

The occasional finding of ‘milking’ of the first septal branch and the anterior descending artery (LAD) on coronarography allowed the ‘open’ biopsy to be combined with myocardial bridge unroofing.

Macroscopically, both myocardial bridge and ventricular mass were indistinguishable; consistent with the literature, the definitive diagnosis of HMCM was histological. It must be emphasized how little is known about this type hamartoma: in a recent report, an atrial HMCM had a high SUV (max 11),^11^ while in a case of HCMC localized in the LV, a low uptake of the radioactive tracer was demonstrated.^12^

## Supplementary Material

ytad077_Supplementary_Data

## Lead author biography

Giacomo Bianchi is a cardiac surgeon at the Heart Hospital in Massa. He focuses on endoscopic valve surgery and surgical therapy of heart failure, including short- and long-term mechanical assists to the circulation. He was a fellow of the Artificial Organs Lab at the University of Maryland in Baltimore for the development of circulatory supports and cell therapy of heart failure. He received a PhD from the Scuola Superiore Sant'Anna in Pisa with experimental work on gene therapy in thoracic aortic aneurysms.

## Supplementary material

**Slide sets:** A fully edited slide set detailing this case and suitable for local presentation is available online as Supplementary data.

**Consent:** The patient has been informed about the present work and has signed an informed consent in accordance with COPE guidelines.

**Funding:** No funding was used for this paper.
