# Supplementary material for: Hamartoma of mature cardiomyocytes presenting with atypical angina, 18F-fluorodeoxyglucose positron emission tomography uptake, and myocardial bridging: a case report
Source: Eur Heart J Case Rep. 2023 Feb 14;7(3):ytad077. doi: 10.1093/ehjcr/ytad077 (PMC9991065; doi:10.1093/ehjcr/ytad077)
Supplement: ytad077_Supplementary_Data [file ytad077_supplementary_data.pptx]

## Slide 1
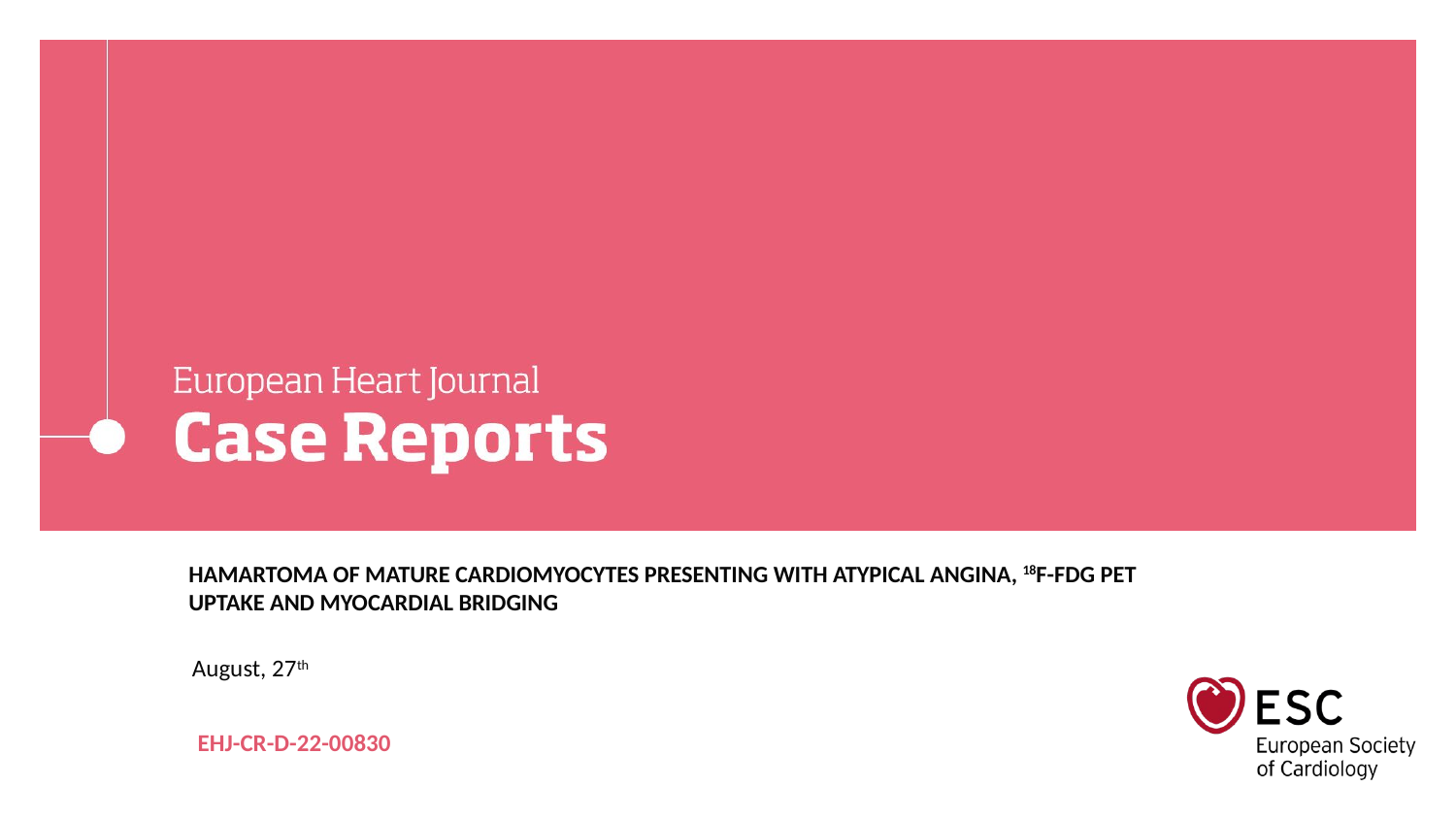

# HAMARTOMA OF MATURE CARDIOMYOCYTES PRESENTING WITH ATYPICAL ANGINA, 18F-FDG PET UPTAKE AND MYOCARDIAL BRIDGING
August, 27th
 EHJ-CR-D-22-00830

## Slide 2
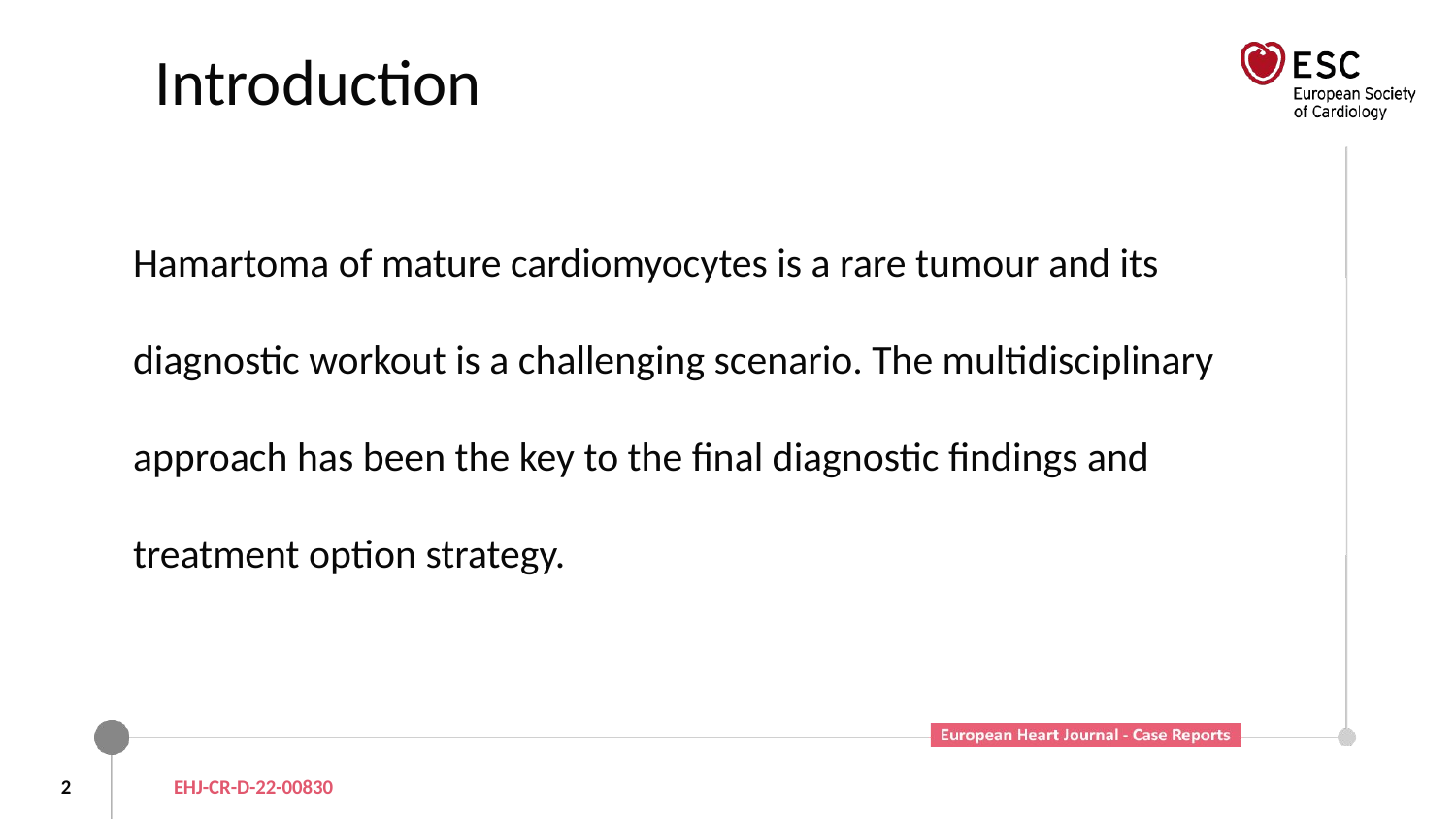

# Introduction
Hamartoma of mature cardiomyocytes is a rare tumour and its diagnostic workout is a challenging scenario. The multidisciplinary approach has been the key to the final diagnostic findings and treatment option strategy.
2
 EHJ-CR-D-22-00830

## Slide 3
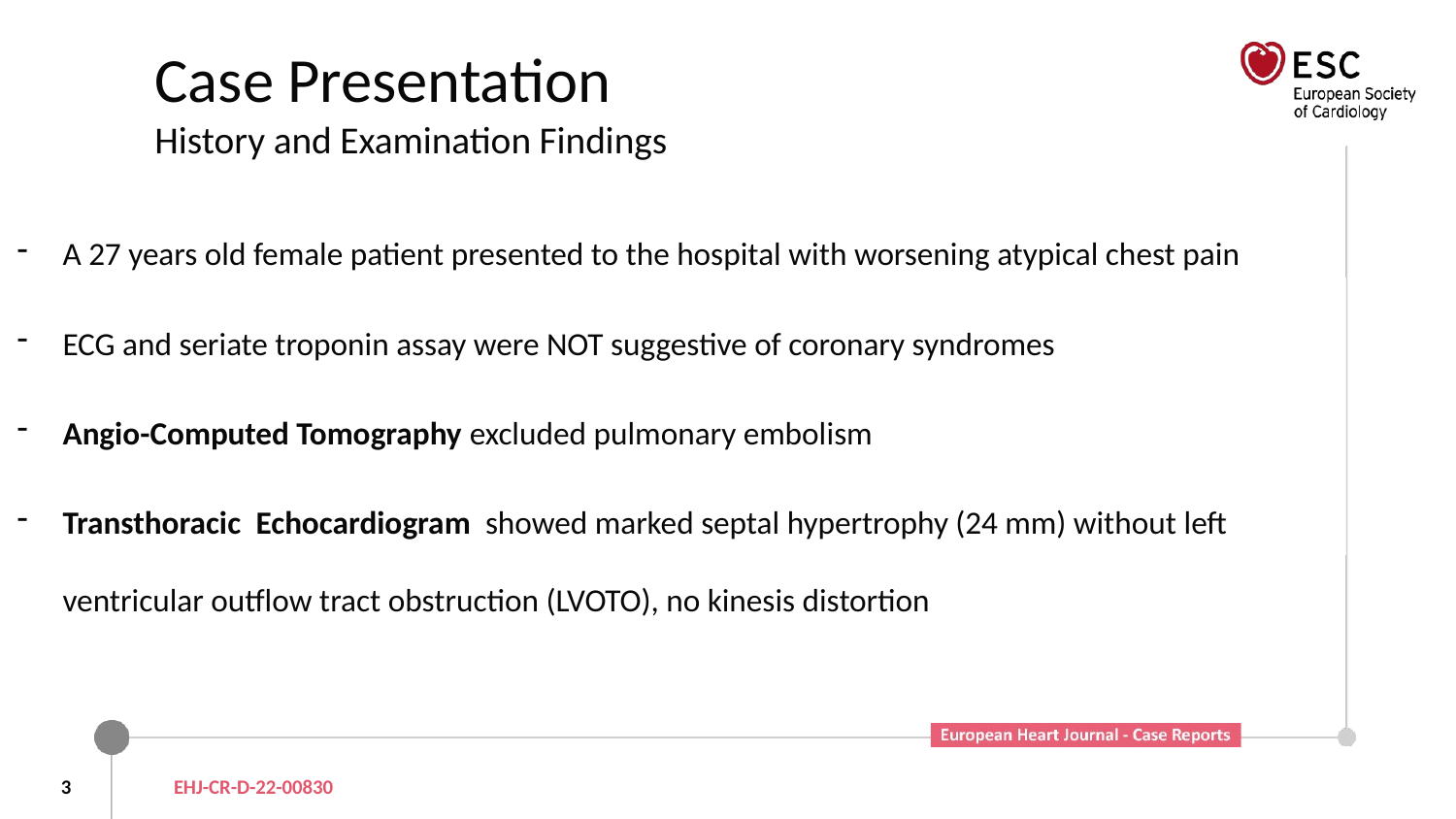

# Case Presentation History and Examination Findings
A 27 years old female patient presented to the hospital with worsening atypical chest pain
ECG and seriate troponin assay were NOT suggestive of coronary syndromes
Angio-Computed Tomography excluded pulmonary embolism
Transthoracic Echocardiogram showed marked septal hypertrophy (24 mm) without left ventricular outflow tract obstruction (LVOTO), no kinesis distortion
3
 EHJ-CR-D-22-00830

## Slide 4
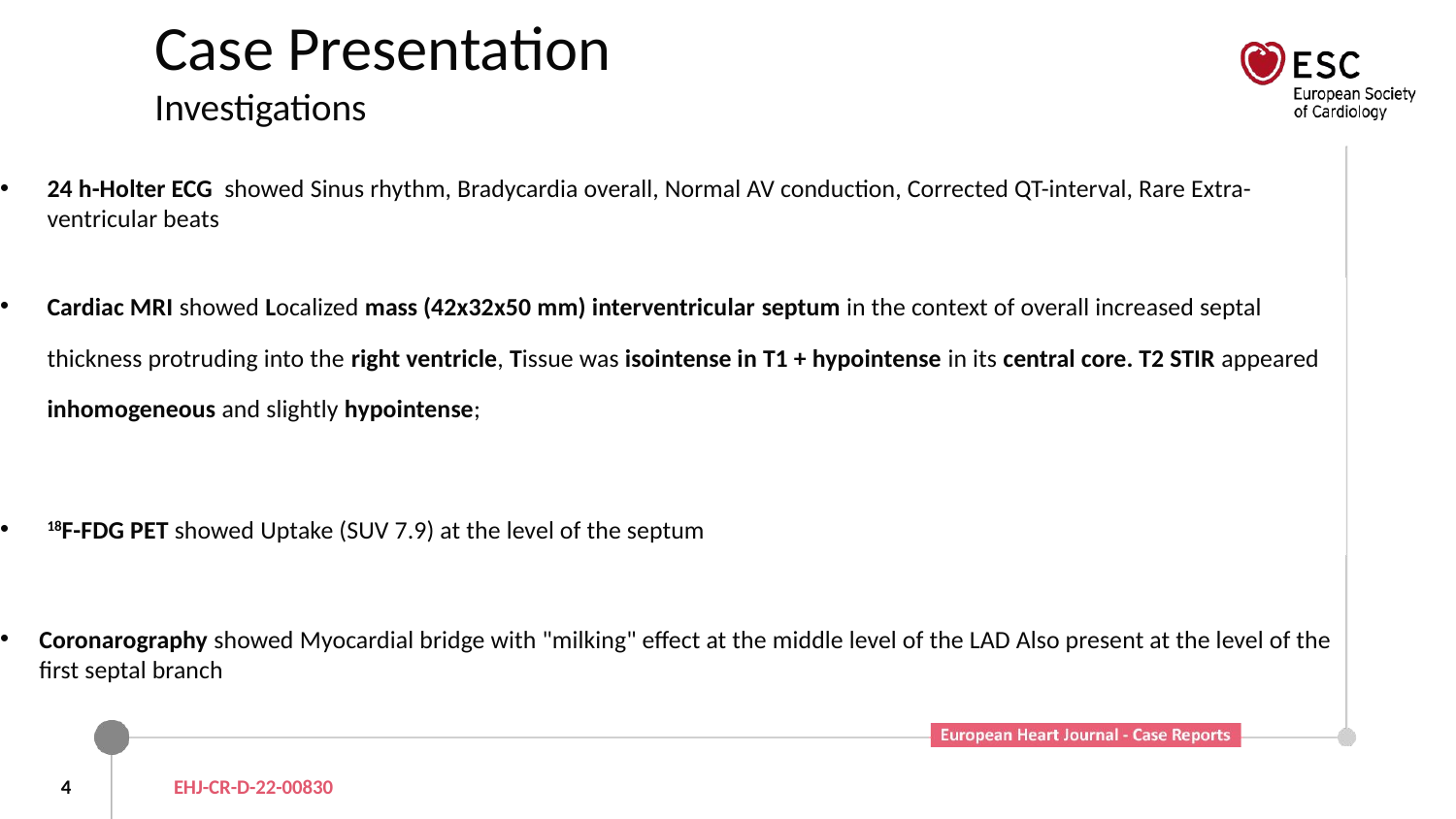

# Case Presentation Investigations
24 h-Holter ECG showed Sinus rhythm, Bradycardia overall, Normal AV conduction, Corrected QT-interval, Rare Extra-ventricular beats
Cardiac MRI showed Localized mass (42x32x50 mm) interventricular septum in the context of overall increased septal thickness protruding into the right ventricle, Tissue was isointense in T1 + hypointense in its central core. T2 STIR appeared inhomogeneous and slightly hypointense;
18F-FDG PET showed Uptake (SUV 7.9) at the level of the septum
Coronarography showed Myocardial bridge with "milking" effect at the middle level of the LAD Also present at the level of the first septal branch
4
 EHJ-CR-D-22-00830

## Slide 5
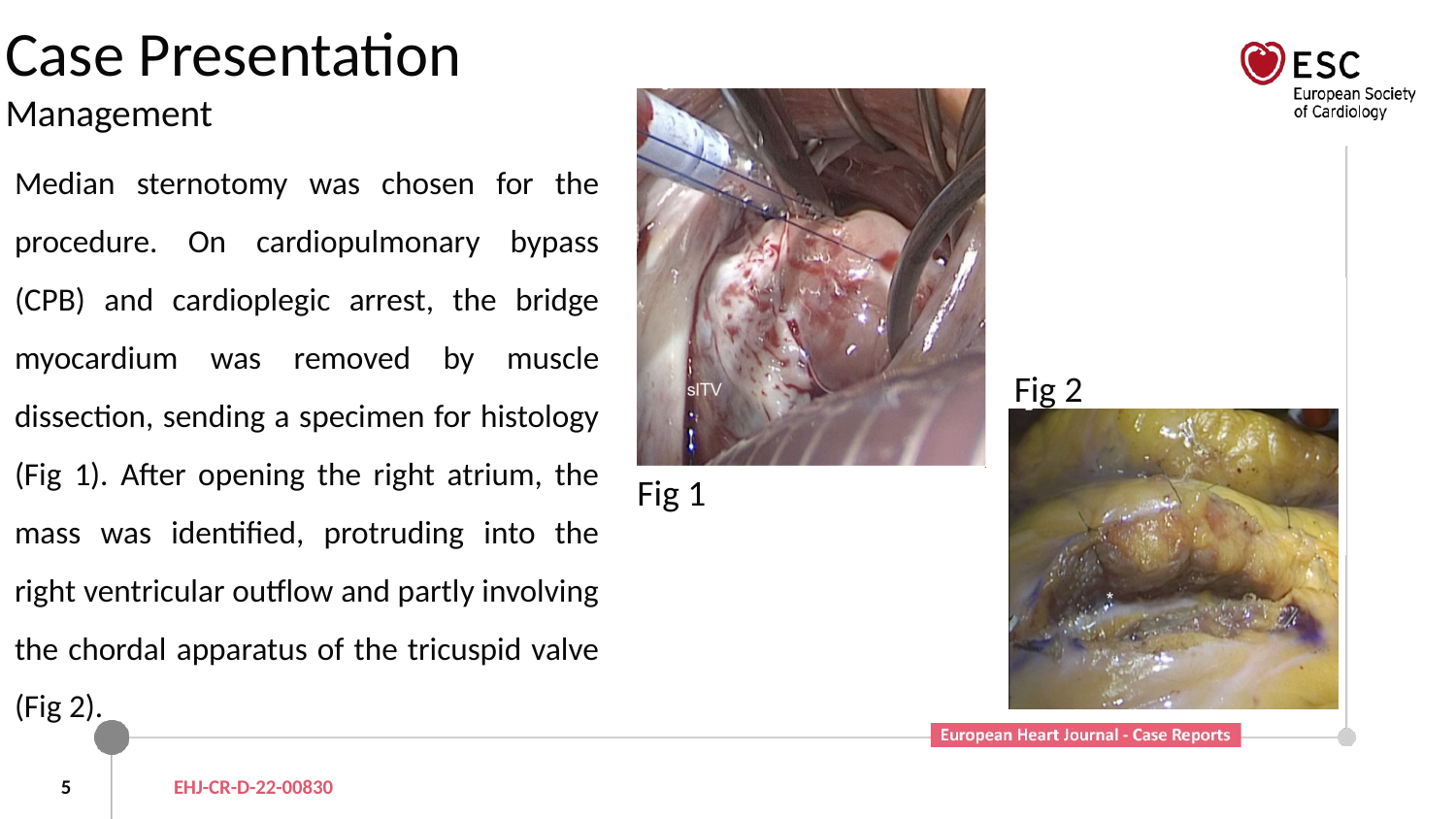

# Case Presentation Management
Median sternotomy was chosen for the procedure. On cardiopulmonary bypass (CPB) and cardioplegic arrest, the bridge myocardium was removed by muscle dissection, sending a specimen for histology (Fig 1). After opening the right atrium, the mass was identified, protruding into the right ventricular outflow and partly involving the chordal apparatus of the tricuspid valve (Fig 2).
Fig 2
Fig 1
5
 EHJ-CR-D-22-00830

## Slide 6
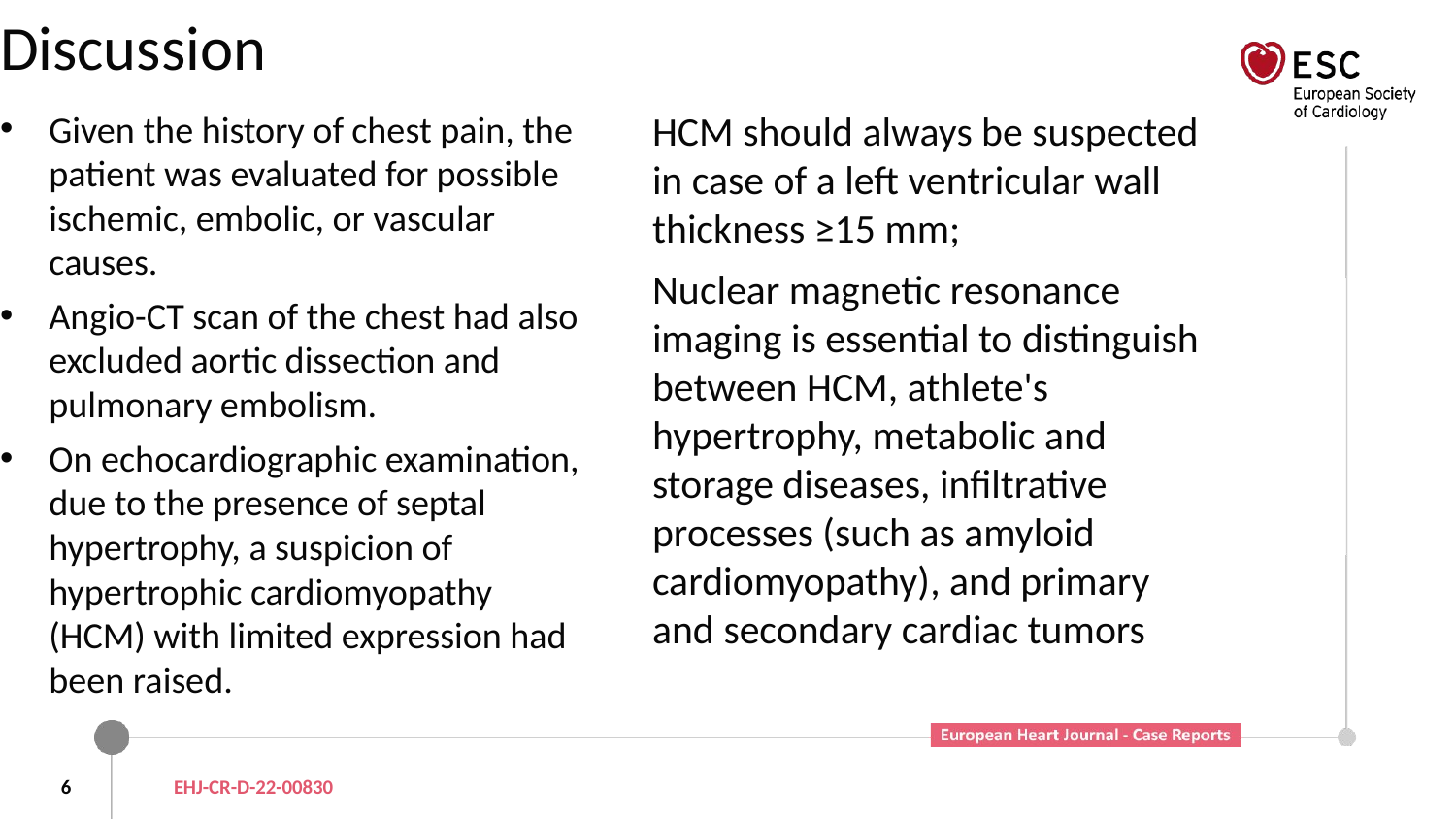

# Discussion
Given the history of chest pain, the patient was evaluated for possible ischemic, embolic, or vascular causes.
Angio-CT scan of the chest had also excluded aortic dissection and pulmonary embolism.
On echocardiographic examination, due to the presence of septal hypertrophy, a suspicion of hypertrophic cardiomyopathy (HCM) with limited expression had been raised.
HCM should always be suspected in case of a left ventricular wall thickness ≥15 mm;
Nuclear magnetic resonance imaging is essential to distinguish between HCM, athlete's hypertrophy, metabolic and storage diseases, infiltrative processes (such as amyloid cardiomyopathy), and primary and secondary cardiac tumors
6
 EHJ-CR-D-22-00830

## Slide 7
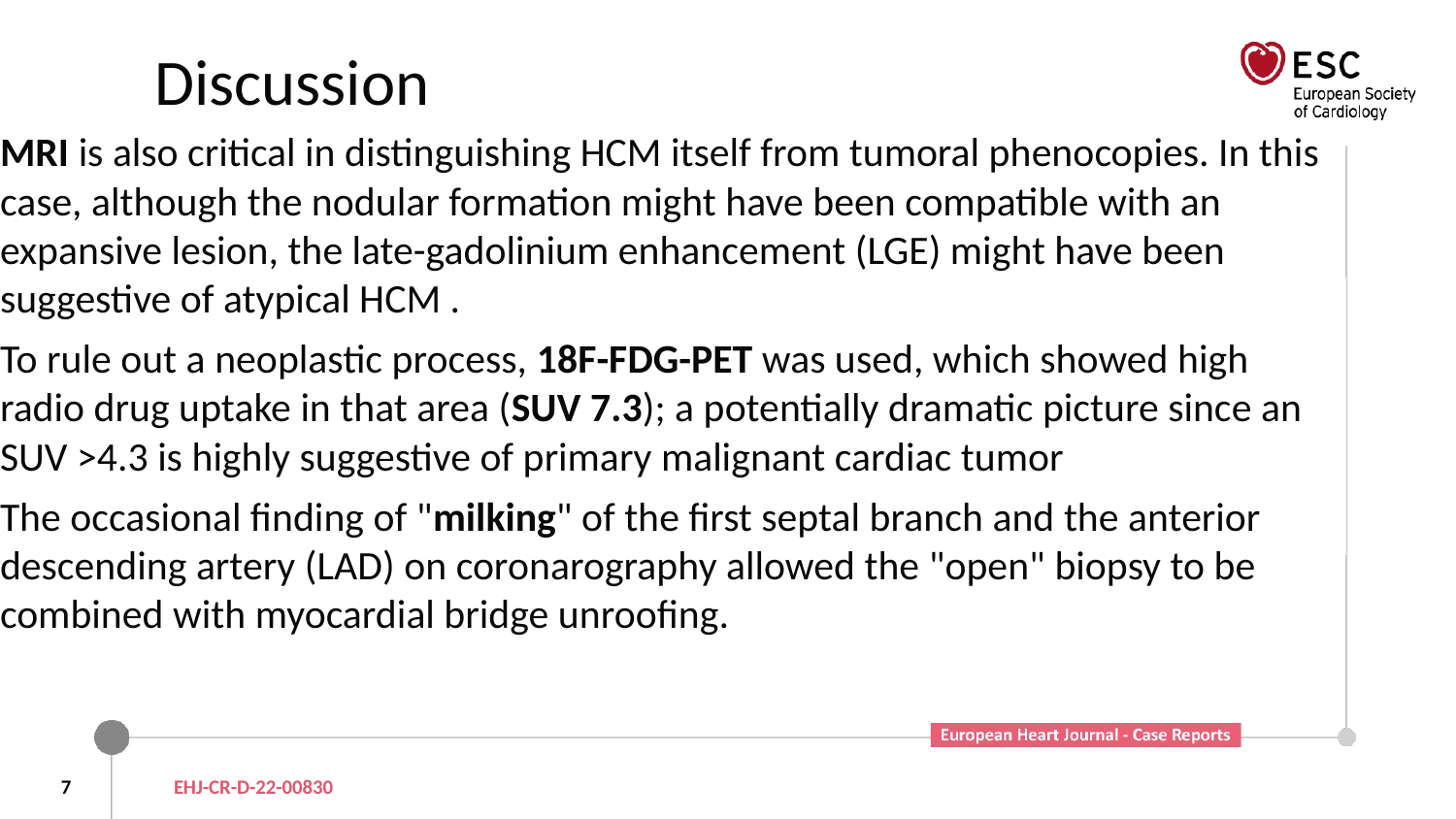

# Discussion
MRI is also critical in distinguishing HCM itself from tumoral phenocopies. In this case, although the nodular formation might have been compatible with an expansive lesion, the late-gadolinium enhancement (LGE) might have been suggestive of atypical HCM .
To rule out a neoplastic process, 18F-FDG-PET was used, which showed high radio drug uptake in that area (SUV 7.3); a potentially dramatic picture since an SUV >4.3 is highly suggestive of primary malignant cardiac tumor
The occasional finding of "milking" of the first septal branch and the anterior descending artery (LAD) on coronarography allowed the "open" biopsy to be combined with myocardial bridge unroofing.
7
 EHJ-CR-D-22-00830

## Slide 8
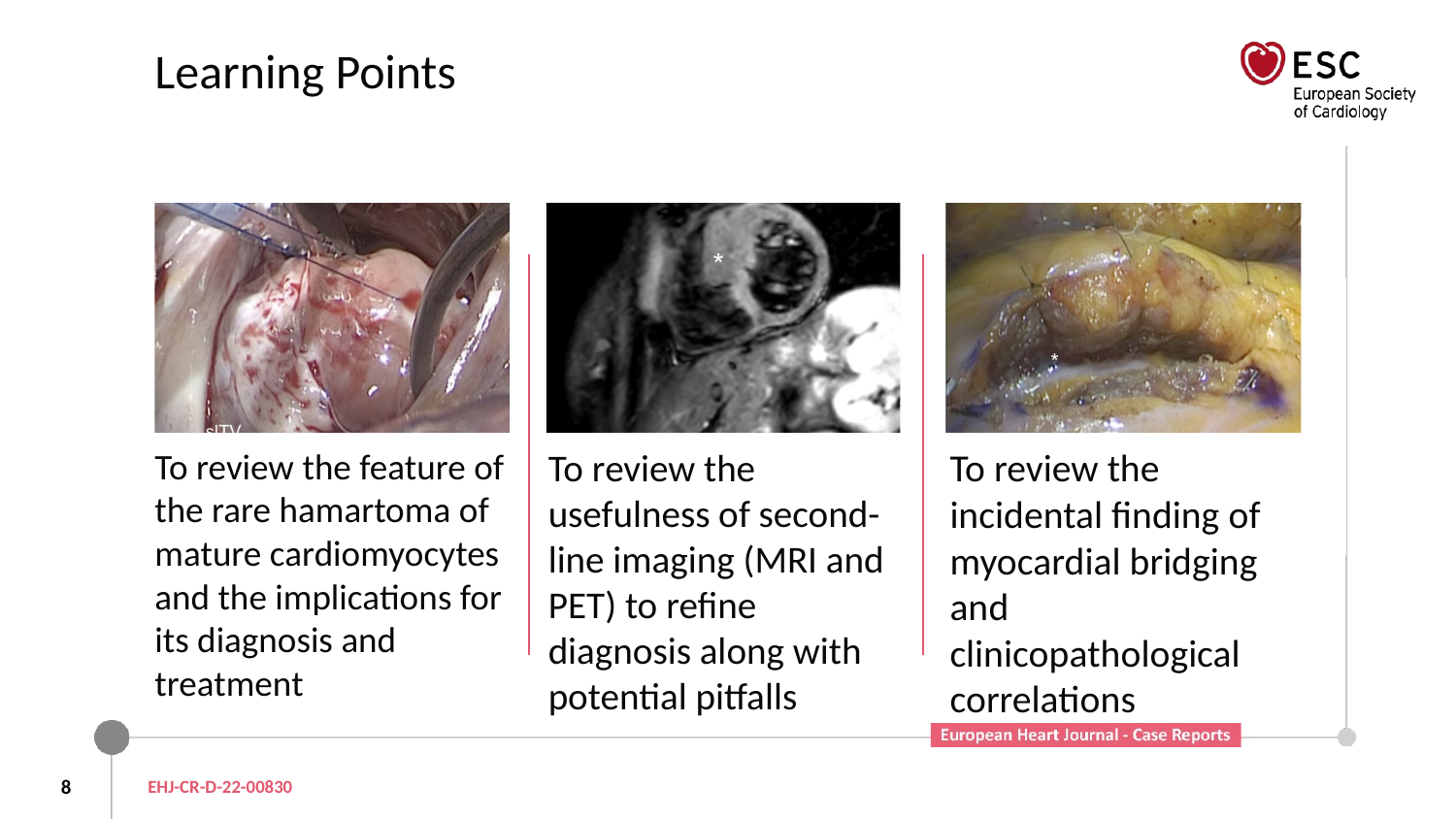

# Learning Points
To review the usefulness of second-line imaging (MRI and PET) to refine diagnosis along with potential pitfalls
To review the feature of the rare hamartoma of mature cardiomyocytes and the implications for its diagnosis and treatment
To review the incidental finding of myocardial bridging and clinicopathological correlations
 EHJ-CR-D-22-00830
8

## Slide 9
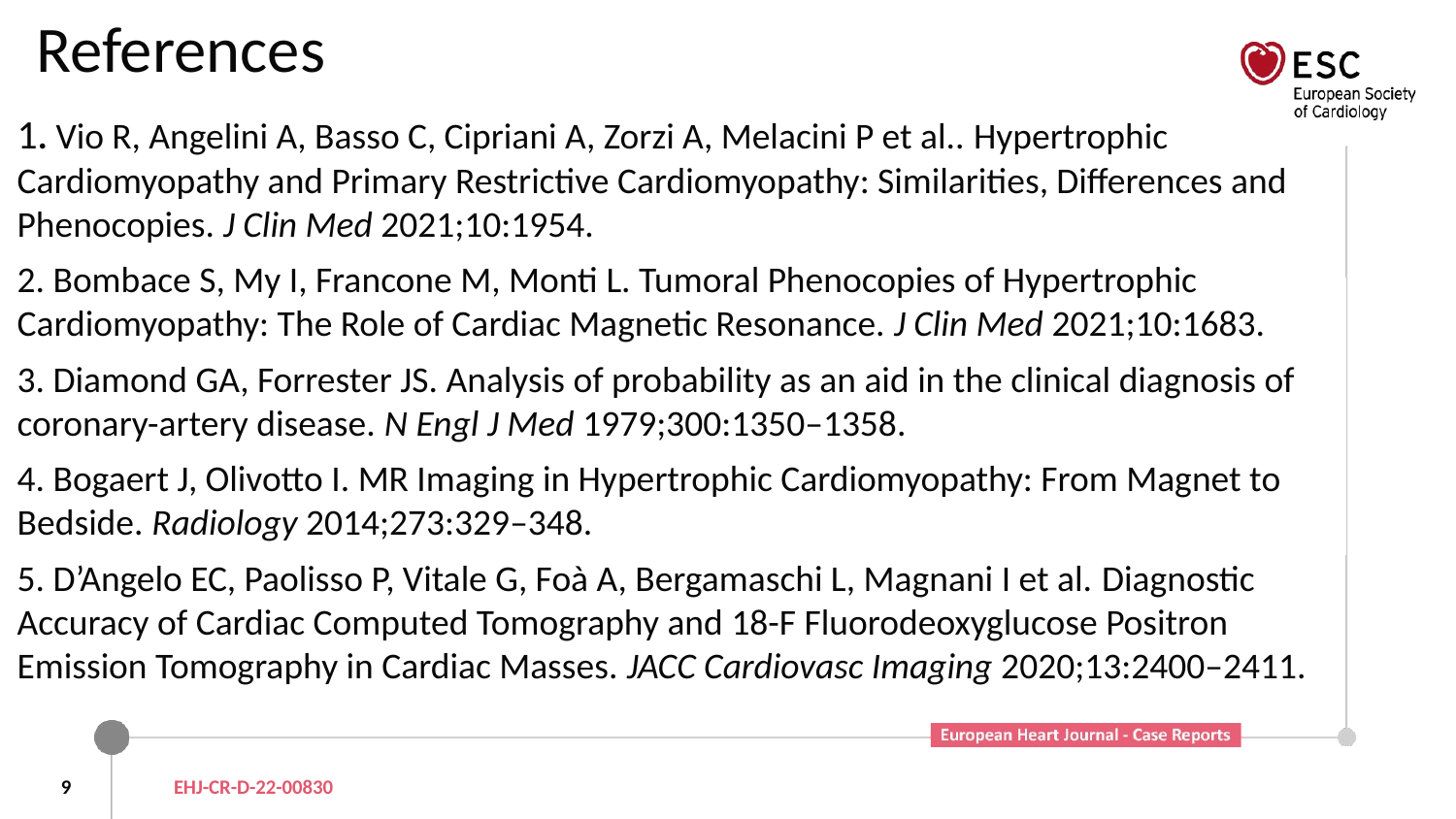

# References
1. Vio R, Angelini A, Basso C, Cipriani A, Zorzi A, Melacini P et al.. Hypertrophic Cardiomyopathy and Primary Restrictive Cardiomyopathy: Similarities, Differences and Phenocopies. J Clin Med 2021;10:1954.
2. Bombace S, My I, Francone M, Monti L. Tumoral Phenocopies of Hypertrophic Cardiomyopathy: The Role of Cardiac Magnetic Resonance. J Clin Med 2021;10:1683.
3. Diamond GA, Forrester JS. Analysis of probability as an aid in the clinical diagnosis of coronary-artery disease. N Engl J Med 1979;300:1350–1358.
4. Bogaert J, Olivotto I. MR Imaging in Hypertrophic Cardiomyopathy: From Magnet to Bedside. Radiology 2014;273:329–348.
5. D’Angelo EC, Paolisso P, Vitale G, Foà A, Bergamaschi L, Magnani I et al. Diagnostic Accuracy of Cardiac Computed Tomography and 18-F Fluorodeoxyglucose Positron Emission Tomography in Cardiac Masses. JACC Cardiovasc Imaging 2020;13:2400–2411.
9
 EHJ-CR-D-22-00830
